# Simultaneous Enhancement of Lithium Transfer Kinetics and Structural Stability in Dual-Phase TiO_2_ Electrodes by Ruthenium Doping

**DOI:** 10.1021/acsami.3c15122

**Published:** 2024-02-08

**Authors:** Jie Zheng, Rui Xia, Najma Yaqoob, Payam Kaghazchi, Johan E. ten Elshof, Mark Huijben

**Affiliations:** † MESA+ Institute for Nanotechnology, University of Twente, P.O. Box 217, Enschede 7500AE, The Netherlands; ‡ Institute of Energy and Climate Research, Materials Synthesis and Processing (IEK-1), Forschungszentrum Jülich GmbH, Jülich 52425, Germany

**Keywords:** dual-phase TiO_2_, ruthenium doping, lithium ion diffusion, structure stability, lithium-ion batteries

## Abstract

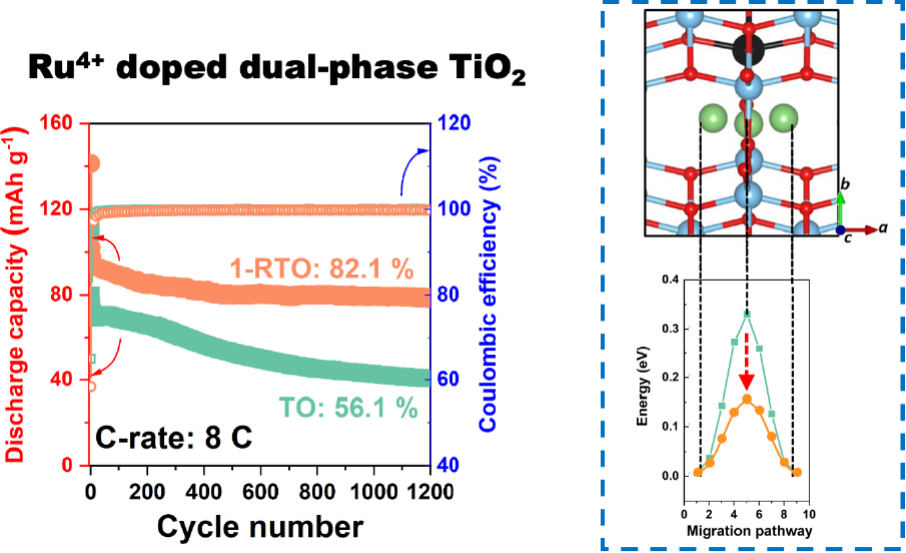

Dual-phase TiO_2_ consisting of bronze and anatase phases is an attractive electrode material for fast-charging lithium-ion batteries due to the unique phase boundaries present. However, further enhancement of its lithium storage performance has been hindered by limited knowledge on the impact of cation doping as an efficient modification strategy. Here, the effects of Ru^4+^ doping on the dual-phase structure and the related lithium storage performance are demonstrated for the first time. Structural analysis reveals that an optimized doping ratio of Ru:Ti = 0.01:0.99 (1-RTO) is vital to maintain the dual-phase configuration because the further increment of Ru^4+^ fraction would compromise the crystallinity of the bronze phase. Various electrochemical tests and density functional theory calculations indicate that Ru^4+^ doping in 1-RTO enables more favorable lithium diffusion in the bulk for the bronze phase as compared to the undoped TiO_2_ (TO) counterpart, while lithium kinetics in the anatase phase are found to remain similar. Furthermore, Ru^4+^ doping leads to a better cycling stability for 1-RTO-based electrodes with a capacity retention of 82.1% after 1200 cycles at 8 C as compared to only 56.1% for TO-based electrodes. In situ X-ray diffraction reveals a reduced phase separation in the lithiated anatase phase, which is thought to stabilize the dual-phase architecture during extended cycling. The simultaneous enhancement of rate ability and cycling stability of dual-phase TiO_2_ enabled by Ru^4+^ doping provides a new strategy toward fast-charging lithium-ion batteries.

## 1 Introduction

The fast charging ability of lithium-ion batteries (LIBs) is of significant importance to many practical applications, such as portable devices and electric vehicles, because charging time can be dramatically reduced with increasing charging power.^1^ Serving as a crucial component in LIBs, the anode material should allow efficient ion and electron transfer to achieve a fast charging process. To avoid the risk of lithium plating at high charging rates, titanium dioxide (TiO_2_) has been proposed because of its safe operating potential (above 1 V vs Li/Li^+^) as well as its stable and open crystal structure for Li^+^ transfer.^2,3^ However, the practical application of TiO_2_ is mainly limited due to its poor ionic and electronic conductivity.^4−6^ Interestingly, TiO_2_ possesses various polymorphs, such as bronze, anatase, and rutile, presenting distinct differences in Li^+^ storage behavior.^7−11^ For instance, full lithiation in anatase TiO_2_ (*x* = 1 in Li*_x_*TiO_2_) can be achieved by nanostructuring. However, only *x* = 0.5 Li^+^ ions can be stored in microsized anatase TiO_2_, leading to a theoretical capacity of 168 mA h g^–1^.^12^ On the other hand, the insertion of Li^+^ in the bronze phase TiO_2_ shows less particle size dependence as the intercalation of *x* = 0.85 and *x* = 1.01 Li^+^ has been demonstrated for micro- and nanoscale particles, respectively.^13,14^

Therefore, constructing nanostructures and nanocomposites incorporating additional conductive materials are the most widely utilized methods to enhance the ion/electron transfer in TiO_2_.^15−22^ Furthermore, it has been recently demonstrated that dual-phase TiO_2_ is able to provide additional diffusion channels and active storage sites at the interfaces between two TiO_2_-based phases.^23−26^ The underlying mechanism is based on the so-called “job-sharing” mechanism, as proposed by Maier et al.,^27,28^ which assumes that the interfaces consisting of a Li^+^-accepting phase and an electron-accepting phase are favorable for extra lithium storage. According to the charge separation argument in semiconductive bronze/anatase TiO_2_,^29^ bronze TiO_2_ can serve as the Li^+^-accepting phase, while anatase serves as the electron acceptor. Therefore, constructing novel architectures of bronze/anatase TiO_2_ appears to be a promising strategy to optimize the lithium storage performance of TiO_2_. However, dual-phase TiO_2_ still suffers from poor ion and electron transfer within the individual phases. Though nanosizing seems attractive, it will inevitably compromise the volumetric energy and power densities because of the low volumetric density and high surface energy of nanomaterial-based electrodes. Sufficiently high volumetric densities require the use of dual-phase TiO_2_ with microsized domains. To enhance the ion/electron transfer within those domains, cation/anion substitution/doping is one of the most popular methods because it can effectively change the local electronic configuration, widen the diffusion channels, as well as tune the system entropy.^30−32^ To date, most studies that optimize the lithium storage performance of dual-phase TiO_2_ by cation substitution and doping are still focusing on nanomaterials. For example, Opra et al.^33^ reported the effect of multivalent vanadium doping to improve the rate and cycling performance of bronze/anatase TiO_2_ nanotubes. Cu^2+^ and Nb^5+^ doping have been demonstrated to enhance the electronic conductivity and thus the rate ability of bronze/anatase TiO_2_ nanowires and nanoparticles, respectively.^34,35^ However, the exploration of the impact of cation doping in microscale particles of dual-phase TiO_2_ is still very limited, and detailed knowledge on its effect on the ion transfer kinetics in the bronze/anatase phase is lacking.

In this work, mesoporous bronze/anatase TiO_2_ with microsized domains is synthesized, and the effects of ruthenium doping on the dual-phase structure and lithium storage performance are investigated. With the electronic configuration of e_g_^0^t_2g_^4^, the unpaired electrons in Ru^4+^ are expected to modify the electronic configuration of Ti^4+^ and consequently improve the electronic conductivity, which were confirmed by X-ray photoelectron spectroscopy (XPS) and electrochemical impedance spectroscopy (EIS). Besides, because of the larger ionic size of Ru^4+^ (0.62 Å) than that of Ti^4+^ (0.605 Å) and the lower thermal stability of the bronze phase compared to the anatase phase,^6^ Li^+^ diffusion channels are widened more easily in the bronze phase by Ru^4+^ doping as compared to the anatase phase. This contributes to enhanced Li^+^ transfer kinetics in the bronze phase, which is demonstrated by kinetic analysis and density functional theory (DFT) calculations. However, an optimal doping ratio is found to be crucial as a further increase in Ru^4+^ levels is found to decrease the crystallinity of the bronze phase, leading to significant capacity loss in that phase. Importantly, Ru^4+^ doping is able to effectively stabilize the dual-phase structural framework, enabling an enhanced stability with a significantly higher capacity retention as compared to that of the undoped case. The enhanced electrochemical behavior can be attributed to a suppressed phase separation of the lithiated anatase due to Ru^4+^ doping. This simultaneous enhancement of the rate ability and cycling stability of dual-phase TiO_2_ by Ru^4+^ doping provides a new strategy toward fast-charging lithium-ion batteries.

## 2 Experimental Method

### 2.1 Materials Synthesis

The bulk parent crystals of pristine (*x* = 0) and ruthenium doped (*x* = 0.0173 and 0.0346) K_0.8_Ti_1.73-*x*_Ru*_x_*Li_0.27_O_4_ (denoted as KTLO or 1-KTRLO and 2-KTRLO) were synthesized by a solid-state reaction. Stoichiometric amounts of commercial K_2_CO_3_, Li_2_CO_3_, TiO_2_, and RuO_2_ powders (Sigma-Aldrich) were mixed by ball-milling for 48 h and were subsequently annealed under 1000 °C for 20 h. To exchange potassium and lithium, protonation was conducted by dispersing KTLO and KTRLO powders in 2 M HNO_3_ for 3 days. The protonated powders H_1.08_Ti_1.73–*x*_Ru*_x_*O_4_, denoted as HTO (*x* = 0), 1-HTRO (*x* = 0.0173), and 2-HTRO (*x* = 0.0346), were cleaned with dilute water and dried at ambient temperature. Finally, the dried powders were calcined at 450 °C for 1 h in air to obtain dual-phase TiO_2_ (denoted as TO, 1-RTO, and 2-RTO, respectively).

### 2.2 Materials Characterization

The structural properties of all powders were investigated by using X-ray diffraction (XRD, PANalytical X’pert PRO diffractometer with Cu Kα radiation, λ = 0.15406 nm). The surface element states were studied by X-ray photoelectron spectroscopy (XPS, Omicron Nanotechnology Gmbh surface analysis system with a photon energy of 1486.7 eV, Al Kα X-ray source). The bonding properties were characterized using Raman spectroscopy (1000 UV Raman spectrometer with a laser wavelength of 514 nm for measurement). The surface morphologies were characterized by high-resolution scanning electron microscopy (HRSEM, Zeiss Merlin HRSEM). The powders were cut by focused ion beam (FIB, JEOL JLB-4700), and the corresponding elemental mappings were collected by energy dispersive X-ray spectrometry (EDX). The local lattice parameters were investigated by transmission electron microscopy (TEM, JEOL JEM-2800 with a beam voltage of 200 kV). The surface areas and pore sizes were determined by utilizing the Brunauer–Emmett–Teller (BET, Germini Vll of Micromeritics) technique.

### 2.3 Electrochemical Measurement

The working electrodes were fabricated by mixing the TO or RTO powders with super P and polyvinylidene difluoride (PVDF, Mw 27500, Sigma-Aldrich) with a mass ratio of 70:20:10. The active materials were milled with super P in an agate mortar, and the mixtures were subsequently transferred to *N*-methyl pyrrolidone (NMP, ≥99%, Sigma-Aldrich) solution where PVDF was dissolved. Ultrasonication was applied to ensure good dispersion of the mixed powders. The mixed slurry was cast on Cu foil and dried in a vacuum oven at 60 °C for 12 h. The mass loading of active materials was ∼1.0 mg cm^–2^. The half-cells were fabricated in a glovebox where the active materials were combined with lithium metal (99.9%, Sigma-Aldrich) and a glass fiber separator (ECC1-01-0012-B/L). The applied electrolyte was composed of 1 M LiPF_6_ in a 1:1 ratio v/v ethylene carbonate/dimethyl carbonate (Sigma-Aldrich, battery grade). As for the operando XRD cell, the mass ratio of the mixed slurry was changed to 50:40:10 to achieve an enhanced electronic conductivity, and the beryllium window was employed as the current collector. All electrochemical measurements were performed in a galvanostat/potentiostat (VMP-300, Biologic) with EC-Lab software at room temperature using commercial lab-scale cells (TU Delft), while the operando cell was an optical test cell from EL-CELL.

### 2.4 Theoretical Modeling

Spin-polarized DFT calculations were performed using the projector augmented wave (PAW) potential method^36^ implemented in the Vienna *Ab Initio* Simulation Package (VASP) code.^37^ Generalized gradient approximation (GGA) within the scheme of Perdew–Burke–Ernzerhof (PBE)^38^ was used as the basis of the exchange-correlation (*XC*) functional. An unit cell of 1 × 4 × 1 and a Gamma-centered *k*-point mesh of 1 × 1 × 2 for DFT calculations were used. An energy cutoff of 520 eV as well as an electronic and a force convergence criterion of 10^–4^ eV and −2.00 × 10^–2^ eV/, respectively, were applied for DFT and nudged elastic band (NEB) calculations. The diffusion energy barrier was computed using the DFT-NEB method with 7 images along the *c*-direction. To obtain the most favorable configuration of Ru in Li_1_Ti_32_O_64_, the total Coulomb energy (*E*_C_) was calculated for all possible configurations with 1 Ru in 32 Ti sites, namely  = 32 structures with a charge state of 1+ for Li, 3+ for Ru, 4+ for Ti, and 2– for oxygen. Then DFT-PBE calculation was performed on the electrostatically most favorable structure, and the lowest total energy structure was found. Total Coulomb energy calculations on possible combinations were carried out using the so-called *supercell* code.^39^ Atomistic structures were visualized with VESTA program.^40^

To find the most favorable site for a single Li ion in TiO_2_ (modeled by a 1 × 4 × 1-unit cell: Li_1_Ti_32_O_64_), the Li-ion migration was calculated along the *a-*, *b-*, and *c*-directions. The DFT-PBE calculations show that the most favorable pathway for Li-ion migration is along the *c*-direction. Afterward, the DFT-NEB calculations were performed to compute the diffusion energy barrier (*E*_b_) along the *c*-direction.

## 3 Results and Discussion

### 3.1 Structural Characterization

**Figure 1:**
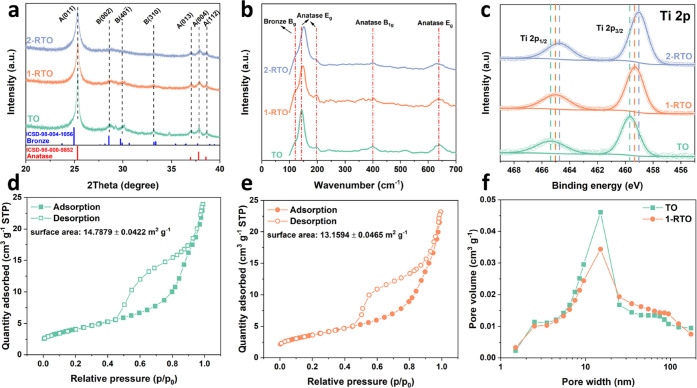
(a) XRD patterns (A = anatase, B = bronze), (b) Raman spectra, and (c) XPS spectra of Ti 2p for TO, 1-RTO, and 2-RTO powders; N_2_ adsorption–desorption isotherms of (d) TO and (e) 1-RTO powders; (f) pore size distributions of TO and 1-RTO powders.

As shown in Figure S1a, XRD patterns of 1-KTRLO and 2-KTRLO exhibit the same diffraction patterns as KTLO with predominantly (020) peaks, demonstrating the typical layered structure of K_0.8_Ti_1.73–x_Ru_*x*_Li_0.27_O_4_ with a highly crystalline lepidocrocite-type crystal structure. After protonation, all three powders displayed identical diffraction patterns in which the (020) peaks had shifted toward lower 2θ angles compared to those in the parent compounds (Figure S1b).The *d*-spacings of the (020) planes are shown in Figure S2, which increased upon protonation by 1.62, 1.58, and 1.44 Å for HTO, 1-HTRO, and 2-HTRO, respectively. The increased *d*-spacing is attributed to the steric effect of the hydration shell of H^+^, which is larger and more coherent than the one surrounding K^+^.^41−43^ Interlayer water and protons are removed at 300 °C, and the formation of the bronze phase starts, followed by the subsequent partial transition from the bronze phase to the anatase phase when the temperature is increased to above 400 °C.^6,23^ As shown in Figure 1a, the XRD patterns of TO and 1-RTO demonstrate a coexistence of bronze and anatase phases. However, with an increasing fraction of Ru^4+^, the peak intensities corresponding to the bronze phase are significantly reduced (2-RTO). It is assumed that the bronze phase has a lower thermal stability than the anatase phase at the specific temperature required to form the bronze/anatase dual phase system. By doping with Ru^4+^, the ionic radius of which is larger than that of Ti^4+^, the crystal structure of the bronze phase tends to become less stable and, therefore, a disordered structure is formed upon increasing the Ru^4+^ dopant concentration. The existence of two phases is further confirmed by Raman analysis (Figure 1b). The TiO_2_ octahedra in the bronze phase cause a shoulder at ∼121 cm^–1^, while in the anatase phase, it leads to a series of peaks with varying vibration modes.^26,44^ It is worth noting that the peaks located at ∼145 cm^–1^ exhibit a blue-shift with increasing Ru^4+^ fraction, implying that a lattice distortion is induced by the substitution of Ti^4+^ by Ru^4+^. The decreased crystallinity of the bronze phase might therefore be ascribed to the lattice-distorting effect of Ru^4+^ as well. Combining XRD and Raman analyses, it is reasonable to conclude that Ru^4+^ is doped into both the bronze and anatase phases. To investigate the effect of Ru^4+^ doping on the electronic configuration of dual-phase TiO_2_, XPS measurements were conducted to characterize the Ti^4+^ state (Figure 1c). Though all three samples exhibit the characteristic peaks of Ti 2p_1/2_ and Ti 2p_3/2_, the obvious peak shifts to lower binding energies indicate that the electron charge density of the titanium ion is increased by unpaired electrons in Ru^4+^ and that a reduced state, i.e., Ti^3+^, is formed.^45^ It is expected that the local electronic configuration of Ru^4+^-doped TiO_2_ is modified and that the electron transfer rate will improve. Based on these considerations, it is clear that the optimal level of Ru^4+^ doping is crucial to enhancing the electrochemical properties as well as maintaining the crystal structure of the bronze phase.

For the sake of comparison, the TO and 1-RTO powders were selected to investigate the effect of Ru^4+^ doping on the bronze/anatase system. The particle size and morphology of TO and 1-RTO were studied by SEM. Both TO and 1-RTO exhibit an irregular particle morphology (Figure S3a,b). As H_3_O^+^ is stored in the interlayer gallery between neighboring Ti_1.73–*x*_Ru*_x_*O_2_ octahedral layers, removal of H_3_O^+^ would result in a topotactic transformation to the TiO_2_ bronze and anatase phases by calcination.^46,47^ The side views of TO and 1-RTO powders illustrate their layered character but with slits (Figure S3c,d). As the lithium ions in the parent compound K_0.8_Ti_1.73–*x*_Ru_*x*_Li_0.27_O_4_ occupy the octahedral sites, they are exchanged upon protonation. The protons are stored between the layers rather than occupying the original octahedral sites, resulting in Ti vacancies in Ti_1.73–*x*_Ru*_x_*O_2_ octahedral layers.^42^ Therefore, internal stresses occur in the octahedral layers and make them bendable. BET analysis was applied to determine the specific surface areas and pore distributions of TO and 1-RTO powders. Figure 1d,e presents the isotherm curves of TO and 1-RTO, respectively, which both correspond to a typical IV adsorption isotherm with a hysteresis loop that suggests a mesoporous pore structure in TO and 1-RTO. The BET surface areas were 14.79 ± 0.04 and 13.16 ± 0.05 m^2^ g^–1^. The pore size distributions of TO and 1-RTO (Figure 1f) show an average pore size of 15 nm, which is in the mesoporous domain. Such a porous structure originates from the removal of water molecules, which results in bending due to internal stress as well as structure transformation into anatase and bronze. It is consistent with a previous study and is expected to accommodate lattice strains occurring during extended cycling when used as electrodes.^6^ In addition, the elemental distribution inside a 1-RTO particle was characterized by EDX mapping. The cross-section of 1-RTO was exposed by FIB cutting (Figure S4a), and the corresponding map shows homogeneous distributions of Ti and Ru (Figure S4b,c).

TEM was applied to further investigate the bronze/anatase dual phase structure of the TO and 1-RTO powders. TEM images in Figure S5a,c show cross-sections where the porous structure of bulk dual-phase TO and 1-RTO particles is clearly observed. The directions of view are perpendicular to the TiO_6_ octahedral layers, which are along the [100] zone axis. The selected area electron diffraction (SAED) patterns that correspond to the selected areas (marked by red circles in Figure S5a,c) exhibit diffraction spots instead of diffraction rings (Figure S5b,d). The combination of bronze and anatase phases in TO and 1-RTO is demonstrated by the presence of diffraction spots of the (004) plane of anatase as well as the (001) plane of bronze. The presence of the anatase phase is further proven by a predominant spot of the (011) plane, which is ∼69° rotated with respect to (004) plane.

**Figure 2:**
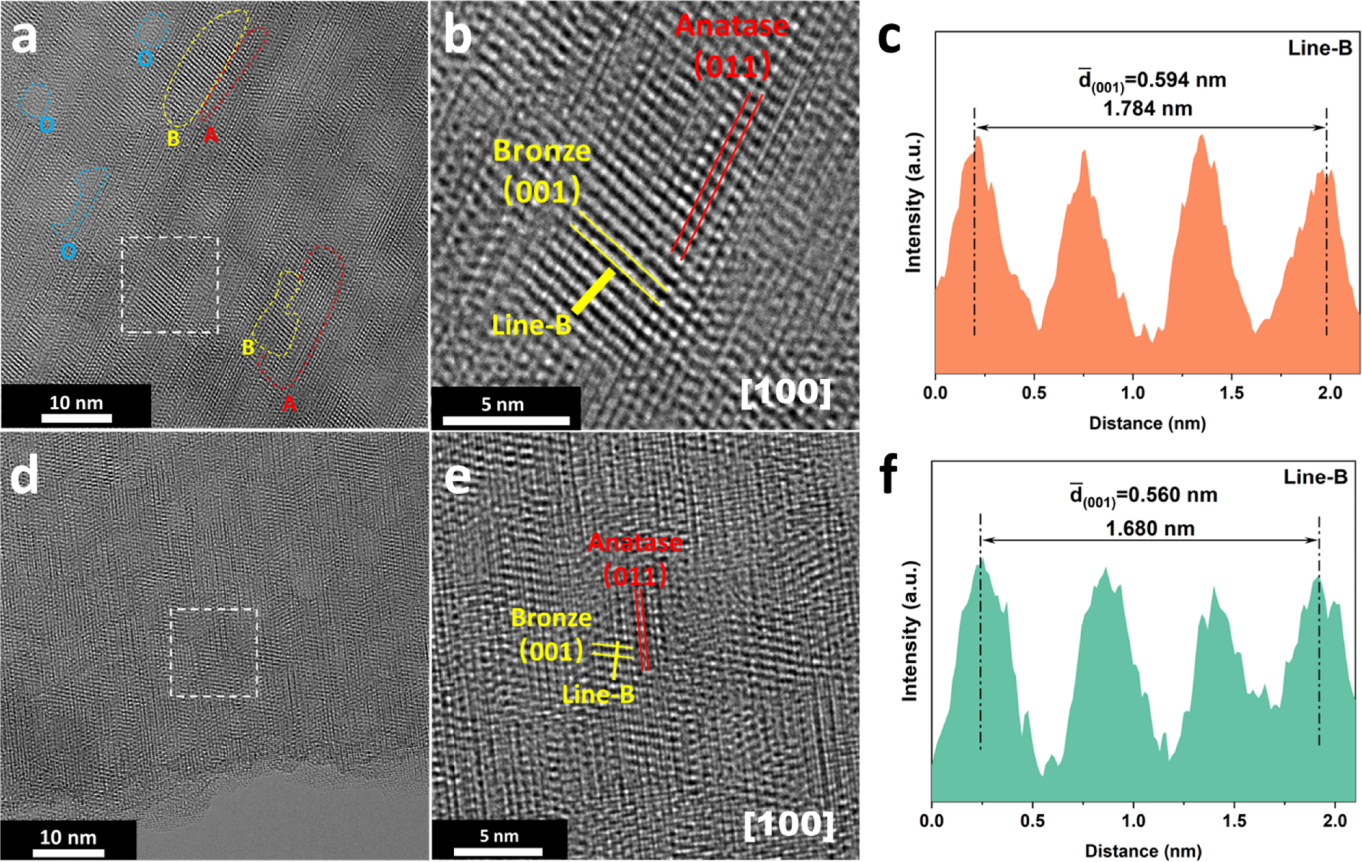
HRTEM images of (a) 1-RTO and (d) TO particles; the zoom-in HRTEM images of (b) 1-RTO and (e) TO particles that correspond to the white box area in (a) and (d), respectively. The intensity line profiles of (c) 1-RTO and (f) TO particles correspond to line-B in parts (b) and (e), respectively.

High-resolution TEM (HRTEM) was conducted to visualize the anatase/bronze domain structure of 1-RTO and TO in detail. Figure 2a shows a dual-phase 1-RTO consisting of nanodomains, which are comprised of bronze (marked with B) and anatase (marked with A). They are separated by porous or disordered regions (marked with O), which were formed after removal of the inserted water molecules and structure transformation. The interfaces between the two phases are shown in Figure 2b. The (001) planes of the bronze phase are connected to the anatase (011) planes. Dual phase TO (Figure 2d,e) exhibits a similar phase boundary construction as 1-RTO and is in good agreement with the SAED patterns (Figure S5b,d). Furthermore, the *d*-spacing of (001) planes of the bronze phase in the specific selected area, noted by , is determined by the intensity line profile. The  of 1-RTO is measured to be 0.594 nm (Figure 2c), while that of TO is determined to be 0.560 nm (Figure 2f). The increment of  demonstrates that Ru^4+^ with a larger ionic size leads to an expansion of the crystal lattice. Due to the low doping concentration and low crystallinity of the bronze phase, a peak shift for bronze is not observed in the XRD pattern (Figure 1a). Ru^4+^ doping-induced lattice expansion in 1-RTO is expected to enhance the Li^+^ diffusivity in the bronze phase, as further discussed in Section 3.2. Next to that, the presence of dual-phase domains with numerous phase boundaries is expected to provide extra Li^+^ storage sites beyond those in the individual TiO_2_ phases.

### 3.2 Electrochemical Characterization

**Figure 3:**
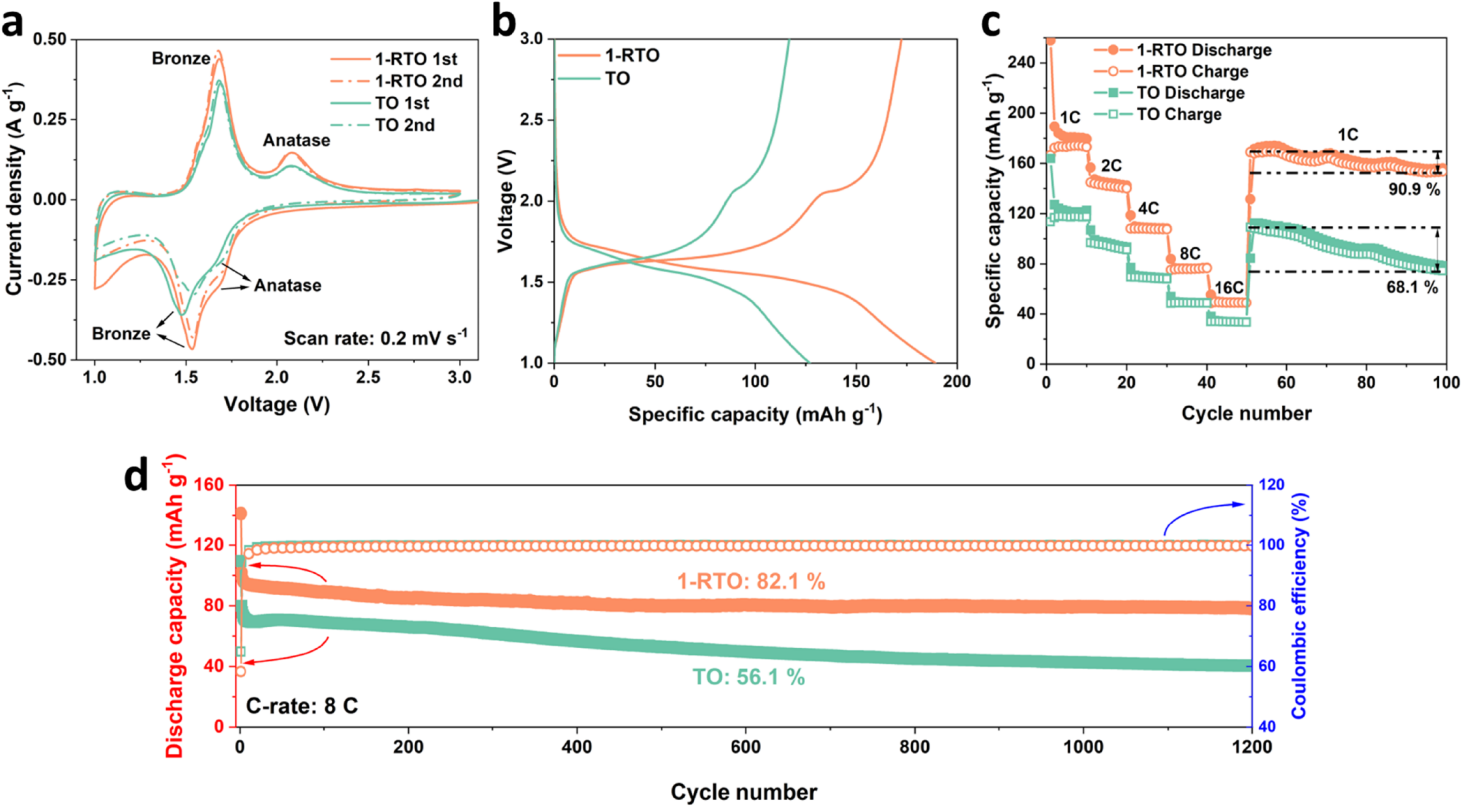
Electrochemical performance of TO- and 1-RTO-based electrodes in a half cell configuration: (a) CV analysis at a sweep rate of 0.2 mV s^–1^; (b) charge–discharge curves during the second cycle; (c) rate performance, and (d) cycling stability.

To investigate the effect of Ru^4+^ doping on the lithium storage performance of the dual-phase TiO_2_ system, lithium-based half-cells were fabricated and analyzed using various electrochemical techniques. Cyclic voltammetry (CV) curves presented in Figure 3a display the redox behaviors of TO- and 1-RTO-based electrodes at a sweep rate of 0.2 mV s^–1^. Both of them show two pairs of redox peaks, which are located within the voltage ranges of 2.1–1.7 V and 1.75–1.5 V, corresponding to (de)lithiation in the anatase and bronze phases, respectively.^7^ Instead of showing separated peaks, as typically observed in other studies, (de)lithiation in bronze phases of TO and 1-RTO occurs in merged peaks because of the low crystallinity of the bronze component.^48^ It is shown that the reduction peak of the bronze phase for 1-RTO remains more stable in the second CV scan as compared to that of TO, which is attributed to the phase stabilization by Ru^4+^ doping and will be discussed in Section 3.3. Besides, it is clear that 1-RTO exhibits a higher current density in both the bronze and anatase peaks than TO, implying more favorable lithiation induced by Ru^4+^ doping. However, a significantly lower peak current density occurs in the bronze phase of 2-RTO (Figure S6), which is attributed to the more disordered and distorted bronze structures resulting from the relatively high Ru^4+^ dopant level. To allow a comparison of material systems with similar crystallinity, the following discussion focuses mainly on TO- and 1-RTO-based electrodes.

Figure S7 shows the charge–discharge curves of TO and 1-RTO at 1 C (1 C = 170 mA g^–1^) for the initial cycle. TO and 1-RTO display discharge/charge capacities of 162.9/113.3 and 256.8/166.5 mA h g^–1^, respectively, corresponding to initial Coulombic efficiencies of 69.5 and 64.8%. The irreversible capacities were previously attributed to the incomplete elimination of surface impurities when annealing hydrogen titanate at 450 °C.^23,49^Figure 3b presents the charge–discharge curves for the second cycles, in which TO and 1-RTO exhibit reversible plateaus in the potential ranges of 2.2–1.6 and 1.6–1.3 V, which are attributed to (de)lithiation in the anatase and bronze phases, respectively, and consistent with CV analysis. 1-RTO was able to deliver a reversible capacity of 172.3 mA h g^–1^, while TO only achieved 116.6 mA h g^–1^. Furthermore, as shown in Figure 3c, 1-RTO delivers higher reversible capacities compared to TO at all tested rates from 1 to 16 C, implying an improved rate ability of dual-phase TiO_2_ with Ru^4+^ doping. When the C-rate was returned to 1 C, the TO and 1-RTO electrodes were subsequently tested by galvanostatic cycling for 50 cycles. Interestingly, the 1-RTO-based electrode exhibited a much better electrochemical stability, demonstrating a high capacity retention of 90.9% as compared to 68.1% for the TO-based electrode. The better cycling stability of 1-RTO was further demonstrated by extended cycling tests at a higher C-rate (Figure 3d). As the initial 5 cycles involved activation of the electrodes, the capacity retentions are calculated based on the capacities for the fifth cycle. In particular, a capacity retention of 82.1% was achieved by 1-RTO after 1200 cycles at 8 C, which is significantly higher than 56.1% for TO. Furthermore, both of them display Coulombic efficiencies above 99.9% between the 5th and 1200th cycles. Based on these observations, it appears that Ru^4+^ doping enables the simultaneous enhancement of the rate ability and cycling stability of bronze/anatase dual phase TiO_2_.

### 3.3 Mechanism Analysis

**Figure 4:**
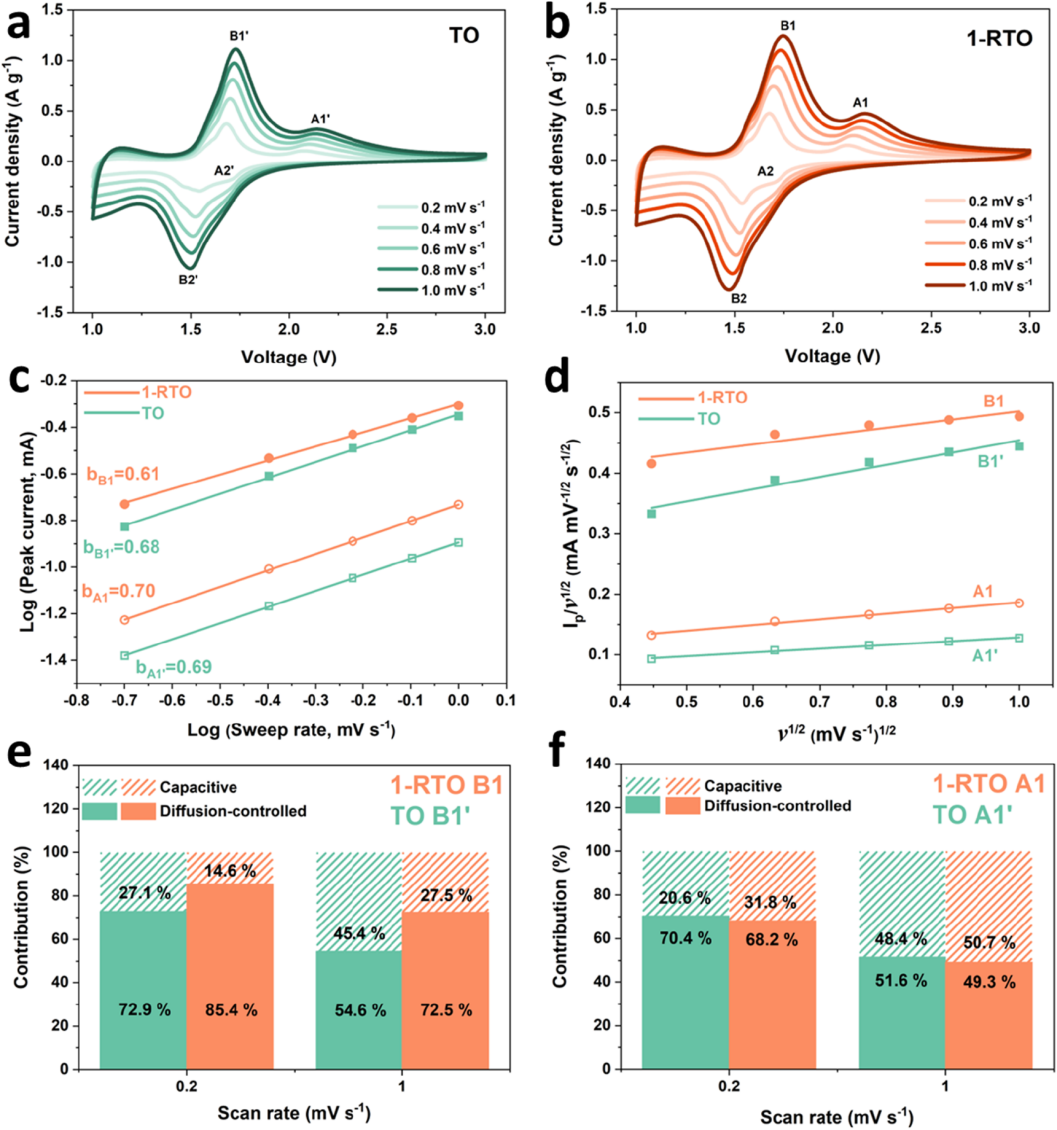
CV curves (a) TO and (b)1-RTO electrodes at various sweep rates; (c) fitted log(*v*)–log(*i*_p_) lines for *b*-value analysis; (d) fitted *I*_p_/*v*^0.5^–*v*^0.5^ lines for *k*_1_ determination. Contribution ratio of diffusion-controlled and capacitive processes at the potential of (e) B1/B1′ and (f) A1/A1′ peak currents at 0.2 and 1.0 mV s^–1^, respectively.

To further study the underlying mechanism of the enhanced rate performance, CV tests at various sweep rates from 0.2 to 1.0 mV s^–1^ were conducted. CV curves of TO and 1-RTO in Figure 4a,b exhibit a series of redox peaks in the potential range of 2.0–1.25 V, which correspond to (de)lithiation behavior in the bronze phase indicated in the figure as B1′/B2′ and B1/B2, respectively. Another group of redox peaks, which can be attributed to the anatase phase in TO and 1-RTO, ismarked as A1′/A2′and A1/A2, respectively. As the lithiation process in the bronze phase is different from that in the anatase phase, the peak currents of all individual peaks should be collected for analysis. However, with the increment of sweep rates, the A2′ and A2 peaks shifted to lower potentials and merged with B2′ and B2, respectively. Therefore, the B1′/A1′ and B1/A1 peaks were selected for further kinetic analysis. According to the following power law relationship,^50^ the *b-*value can be determined from the peak currents (*i*_*p*_) and scan rates (*v*) via:
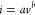
1

The value of *b* is an indication of the storage mechanism of the electrode, where absolute diffusion-limited processes lead to a *b-*value of 0.5, and complete capacitive-dominated behavior results in a *b*-value of 1.0. The fitted log(*i*_p_)–log(*v*) lines, of which the slopes provide the *b*-values, are shown in Figure 4c. A1 and A1′ give similar *b*-values of 0.70 and 0.69, respectively, while B1 gives a smaller *b*-value of 0.61 compared to that of B1′ of 0.68. Though all *b*-values are located in the range of 0.5–1.0, suggesting the cocontributions of diffusive and capacitive effects to the overall currents, the discrepancy between B1 and B1′ requires further analysis. Following Dunn’s model,^51^ the effect of diffusion-controlled and capacitive contribution can be separated using the following equation:
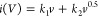
2

It illustrates that the response current (*i*) at a specific potential (*V*) is composed of a capacitive component (*k*_1_*v*) and a diffusion-controlled contribution (*k*_2_*v*^0.5^). Because B1′ and B1 are partially merged with the A1′ and A1 peaks, respectively, only the peak current positions are considered in our analysis. eq 2 can be rearranged to the following linearized equation:
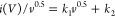
3

To determine *k*_1_, *i*(*V*) at the potential of the peak current and the corresponding sweep rates are plotted in Figure 4d based on eq 3. The specific contributions of diffusion-controlled and capacitive processes to the total peak current positions for B1′/A1′ and B1/A1 were estimated and are presented in Figure 4e,f. It shows that the storage mechanism of the B1 peak is dominated by diffusion-controlled behavior, up to 85.4% and 72.5% at 0.2 and 1.0 mV s^–1^, respectively, and is significantly higher than that of B1′. On the other hand, the degrees of contribution of the diffusion-controlled process to the A1 peak is very close to that of the A1′ peak. Typically, diffusion-controlled processes are related to the bulk intercalation of Li^+^ into the crystal framework, while capacitive behavior involves Li^+^ storage at the surface or at the interface and is faradaic in origin.^52^ Given the fact that the surface areas of TO and 1-RTO are similar (Figure 1d,e), the sites of accommodating Li^+^ in the surface regions are assumed to be similar, as well. In this case, the higher relative contribution of the diffusion-controlled process for the B1 peak indicates that bulk intercalation of Li^+^ in the bronze phase of 1-RTO is able to deliver a higher reversible capacity due to enhanced Li^+^ diffusivity, as further demonstrated in the discussion below.

**Figure 5:**
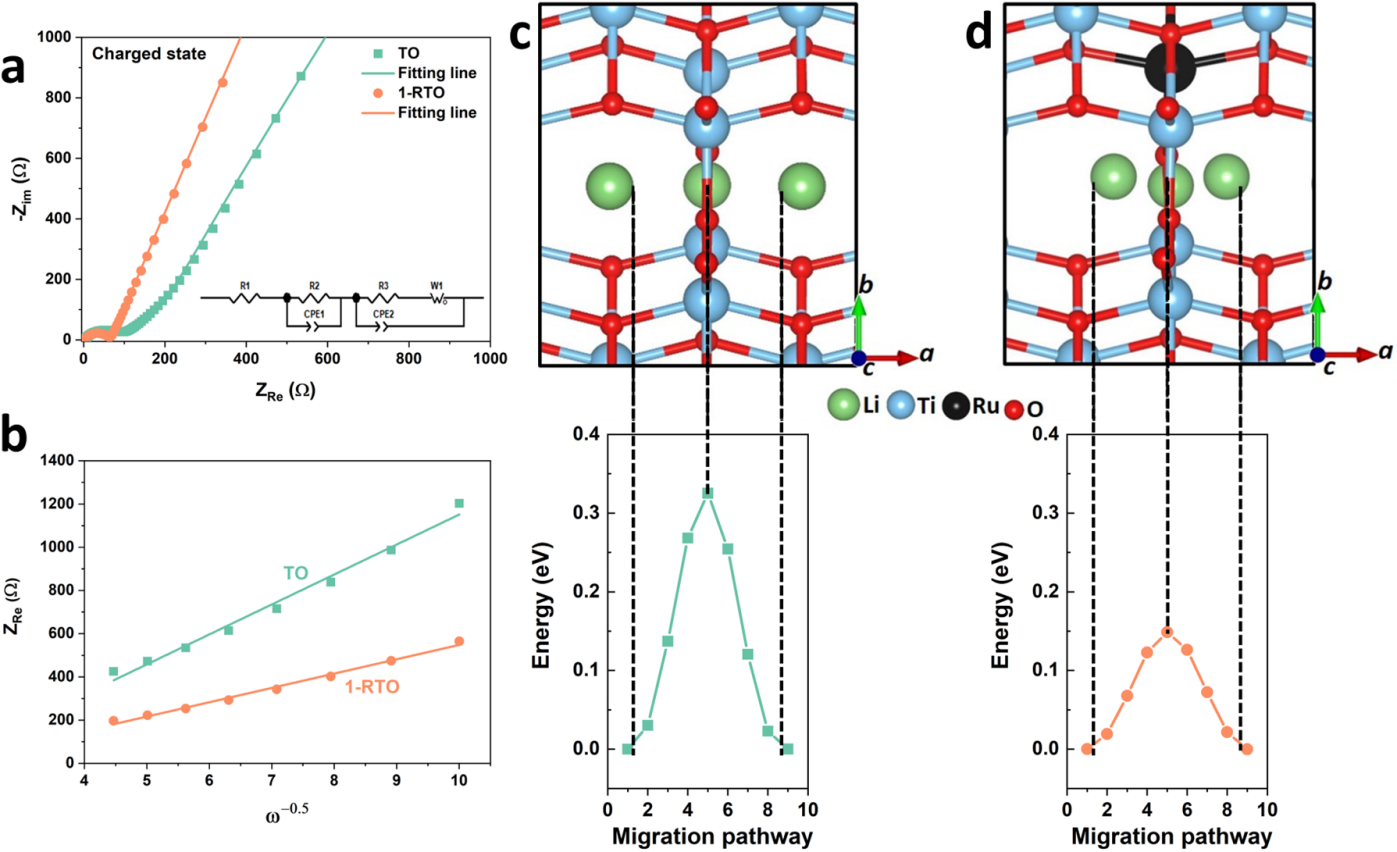
(a) The EIS plots at charged state and the corresponding fitted curves of TO- and 1-RTO-based electrodes, where the equivalent circuit is inserted; (b) fitted *Z*_Re_–ω^–0.5^ lines that correspond to the Warburg region in (a). Calculated Li^+^ diffusion energy barrier of (c) TO and (d) 1-RTO.

The influence of Ru^4+^ doping on the electron and ion transfer in this dual-phase system was explored by electrochemical impedance spectroscopy (EIS) analysis. The Nyquist plots for TO and 1-RTO in their charged states are shown in Figure 5a. The Nyquist plots exhibit a semicircle in the high frequency domain, which is attributed to the electron transfer reaction.^53^ Therefore, an equivalent circuit was proposed to fit the impedance data and predict the charge transfer resistance, which corresponds to the R2 element in Figure 5a. The fitted R2 value for 1-RTO is 62.9 Ω, which is lower than 93.0 Ω for TO, thus pointing to favorable electron transfer kinetics that are probably induced by the unpaired electrons in Ru^4+^. Furthermore, as the Warburg region in the low frequency domain is dominated by the ion diffusion process, it can be utilized to determine the Li^+^ diffusion coefficient based on the following equation:^54^
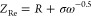
4
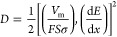
5where *V*_m_ is the molar volume, *F* is the Faraday constant and *S* is the surface area of the electrode that effectively contacts the electrolyte. (d*E/*d*x*) is determined by the first derivative of voltage (*E*) vs composition (*x*). σ is the Warburg factor determined by the linear relation between *Z*_Re_ and ω^*–*0.5^ in eq 4, where *Z*_Re_ is the Warburg impedance in the low frequency (ω) domain. The fitted *Z*_Re_–ω^*–*0.5^ lines in Figure 5b give σ-values of 138.7 and 66.3 for TO and 1-RTO, respectively. Thus, the Li^+^ diffusion coefficients () were calculated to be 3.3 × 10^–15^ and 2.4 × 10^–14^ cm^2^ s^–1^ for TO and 1-RTO, respectively. As discussed in the CV analysis, the diffusion-controlled contribution ratio in the anatase phase of 1-RTO is similar to that of TO. Therefore, it is reasonable to conclude that the enhanced  of 1-RTO originates from the more favorable Li^+^ transfer process in the bronze phase, which is enabled by the larger *d*_(001)_ induced by Ru^4+^ doping (as demonstrated in Figure 2c,f). Density functional theory (DFT) calculations were carried out to investigate the difference in Li^+^ intercalation character in TO and 1-RTO. As shown in Figure 5c,d, the computed diffusion energy barriers of 0.325 and 0.15 eV for nondoped and Ru^4+^-doped bronze TiO_2_, respectively, show that the Li^+^ diffusion kinetics in the Ru^4+^-doped bronze phase are enhanced due to the expansion of the lattice along the *a-* and *b*-directions. The faster Li^+^ diffusion in Ru^4+^-doped bronze is consistent with kinetic analysis based on CV and EIS techniques.

**Figure 6:**
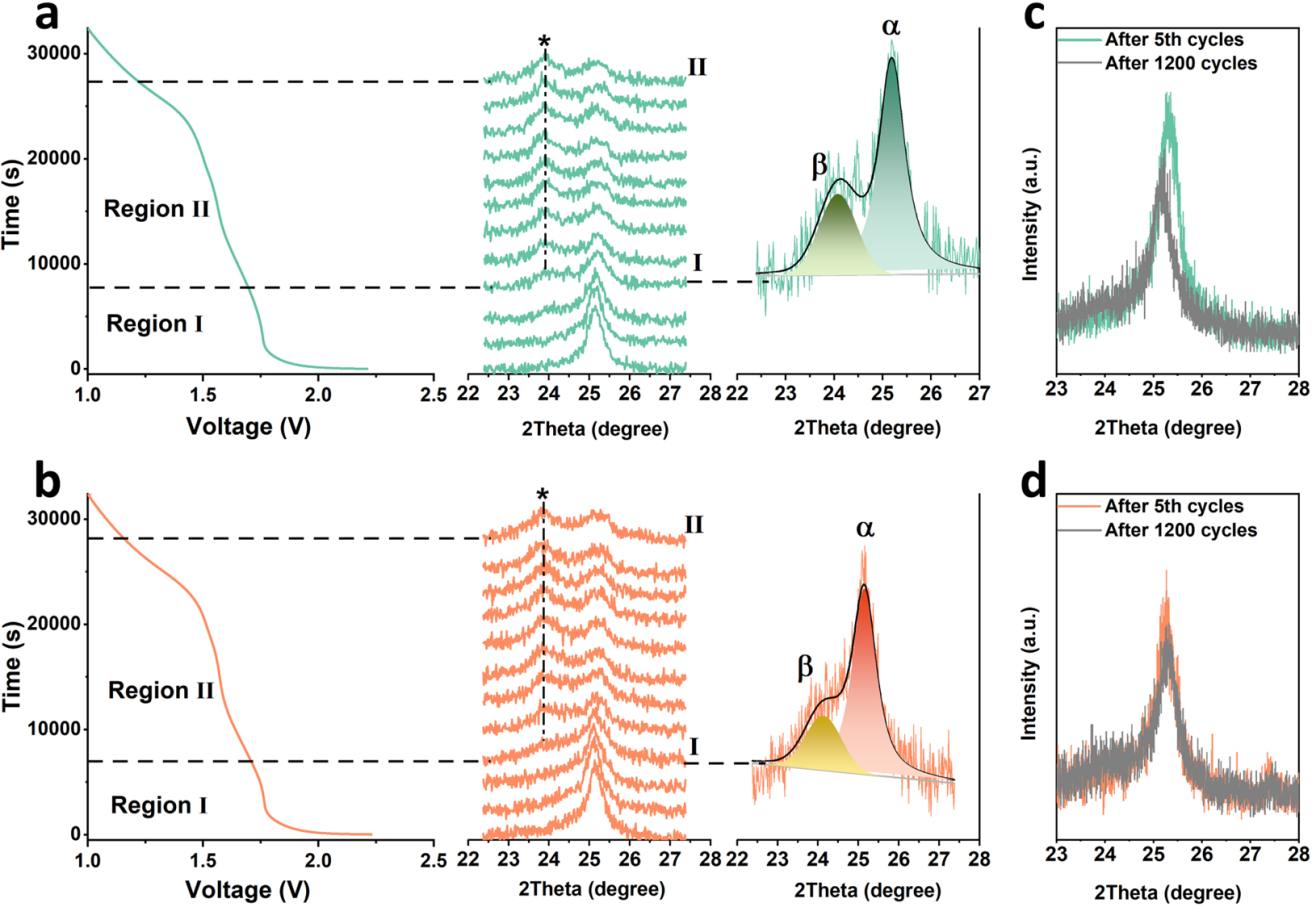
Discharge curves and corresponding in situ XRD patterns of (a) TO- and (b) 1-RTO-based electrodes; comparison of XRD patterns of (c) TO- and (d) 1-RTO-based electrodes after 5th and 1200th cycles.

To understand the underlying mechanism of enhanced cycling stability of 1-RTO, operando XRD analysis was performed to investigate the structural evolution of the TO and 1-RTO particles during the lithiation process. Due to the low crystallinity and the beryllium window in the operando setup, only a predominant peak at ∼25.2° is observed and analyzed. The discharge curves of TO and 1-RTO (Figure 6a,b) are divided into two regions (region I and II), which correspond to the lithiation processes in the anatase and bronze phases, respectively. For the lithiation in the anatase phase, the diffraction patterns of TO and 1-RTO, which are denoted as pattern I, show that a shoulder at lower diffraction angles, assigned to the β-Li titanate phase, develops at the expense of the α-Li*_x_*TiO_2_ phase.^55^ Such type of phase separation is attributed to a very low nucleation barrier for the formation of a phase boundary and a much faster movement of the phase boundary as compared to the relatively sluggish self-diffusion process.^55,56^ Although the division of phases into α and β are observed for both TO and 1-RTO particles, pattern I of 1-RTO exhibits a lower peak intensity ratio (*I*_β_/*I*_α_) of ∼28% than TO (∼38%), suggesting a less favorable formation of the phase boundary in the lithiated anatase phase of 1-RTO. It has been claimed that such phase separation in lithiated anatase is due to the existence of a common plane between α and β phases, and that their misfit is negligible.^57^ Based on this, it is hypothesized that the lattice distortion introduced by Ru^4+^ doping in anatase leads to an increase of the interfacial energy and strain energy, thereby increasing the nucleation barrier for the formation of a phase boundary.

In region II, the merged peaks denoted by an asterisk (*) are the combination of a β-Li titanate phase and a lithiated bronze phase, as the overall intensities of the denoted peaks are higher (patterns I to II). It has been reported that Li^+^ intercalation into the bronze phase involves solid solution behavior with a single phase transformation.^58^ Given the fact that TO and 1-RTO exhibit identical structural evolution in region II, it is concluded that the underlying reason for the enhanced cycling stability of 1-RTO is the suppressed phase invariant of lithiated anatase. XRD patterns were collected after 1200 cycles and presented in Figure 6c,d. It is shown that the predominant peak of 1-RTO at ∼25.2° remains to be stable, while that of TO has clearly decreased in intensity and shifted to a lower 2θ angle, which can probably be assigned to an inactive intermediate phase between β-Li titanate and α-Li*_x_*TiO_2_ phases. This indicates a lower structural stability in the TO induced by phase separation. By comparing the charge–discharge curves after 600 and 1200 cycles (Figure S8), the 1-RTO-based electrode is able to deliver reversible capacities with stable plateaus, while significant degradation and polarization of the plateaus are observed for the TO-based electrode. This further confirms the harmful impact of phase separation in anatase on the stability of the dual-phase TiO_2_ architecture.

## 4 Conclusion

Doping with Ru^4+^ in dual-phase bronze/anatase TiO_2_ has been shown to simultaneously boost the Li^+^ transfer kinetics and stabilize the dual-phase framework during extended cycling. An optimized fraction of Ru^4+^ of ∼1 at. % is crucial to maintain the dual-phase structure. Higher Ru^4+^ content compromises the crystallinity of the bronze phase, albeit with a negligible influence on the anatase phase. This effect can be attributed to the larger ionic radius of Ru^4+^ and the lower thermal stability of bronze as compared to anatase. At the optimal Ru^4+^ doping level, an enlarged *d*-spacing of (001) planes of bronze in 1-RTO was found to effectively boost Li^+^ diffusion in the bronze phase while having a negligible effect on the anatase phase. The DFT calculations and kinetics analysis based on EIS data demonstrate an enhanced Li^+^ diffusion coefficient in the 1-RTO-based electrode with a lower diffusion energy barrier compared to the TO-based electrode. Moreover, Ru^4+^ doping has been proven by operando XRD to suppress the phase separation in lithiated anatase, leading to improved cycling stability. Thus, 1-RTO-based electrodes exhibit a better rate ability and at the same time also a much more stable cycling performance than TO-based electrodes. This simultaneous enhancement of the electrochemical behavior of dual-phase TiO_2_ as a promising electrode material provides a new strategy toward fast-charging lithium-ion batteries.

## Supporting Information Available

The XRD patterns of KTLO/KTRLO and HTO/HTRO powders; the zoomed-in XRD patterns of KTLO/KTRLO and HTO/HTRO powders; the SEM images of TO and 1-RTO powders; the SEM image of 1-RTO powder and its corresponding EDX mapping; the TEM images of TO and 1-RTO powders and their corresponding SAED patterns; the CV curve of 2-RTO electrode; the charge–discharge curves of TO and 1-RTO for initial cycle; charge and discharge curves of 1-RTO and TO electrodes after different cycles (PDF)
