# Supplementary material for: Simultaneous Enhancement of Lithium Transfer Kinetics and Structural Stability in Dual-Phase TiO2 Electrodes by Ruthenium Doping
Source: ACS Appl Mater Interfaces. 2024 Feb 8;16(7):8616–26. doi: 10.1021/acsami.3c15122 (PMC10895577; doi:10.1021/acsami.3c15122)
Supplement: Supplementary file 1 — am3c15122_si_001.pdf [file am3c15122_si_001.pdf]

Supporting Information

Simultaneous Enhancement of Lithium Transfer Kinetics and Structural Stability in Dual-Phase TiO_2 electrodes by Ruthenium Doping

Jie Zheng[a], Rui Xia[a], Najma Yaqoob[a,b], Payam Kaghazchi[a,b], Johan E ten Elshof[a] and Mark Huijben[,a]*

[a] University of Twente, MESA+ Institute for Nanotechnology, P. O. Box 217 7500AE Enschede, the Netherlands.

[b] Forschungszentrum Jülich GmbH, Institute of Energy and Climate Research, Materials Synthesis and Processing (IEK-1)Jülich 52425, Germany.

*Corresponding authors: m.huijben@utwente.nl

Keywords: dual-phase TiO_2, ruthenium doping, lithium ion diffusion, structure stability, lithium-ion batteries

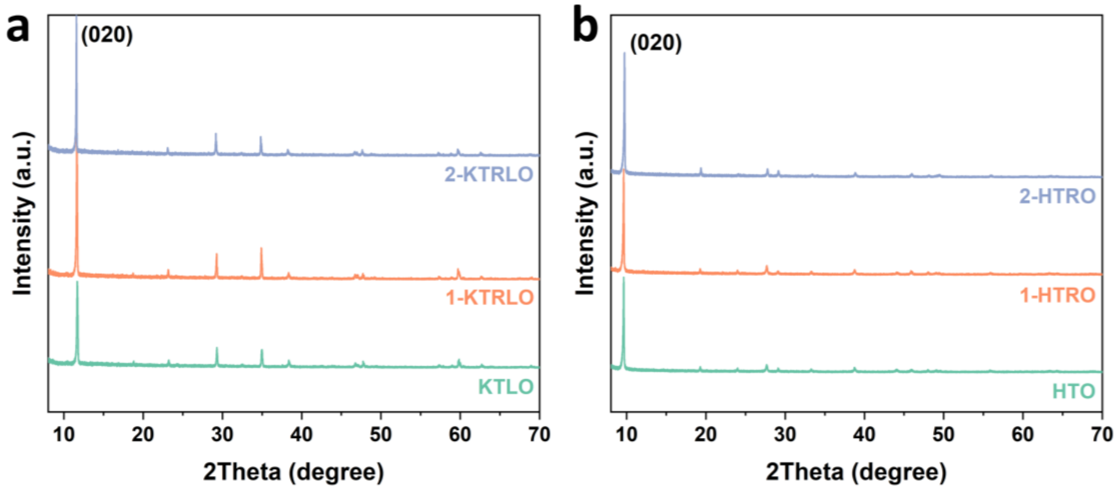

Figure S1. The XRD patterns of (a) initial (KTLO,1-KTRLO,2-KTRLO) and (b) protonated (HTO,1-HTRO,2-HTRO) powders.

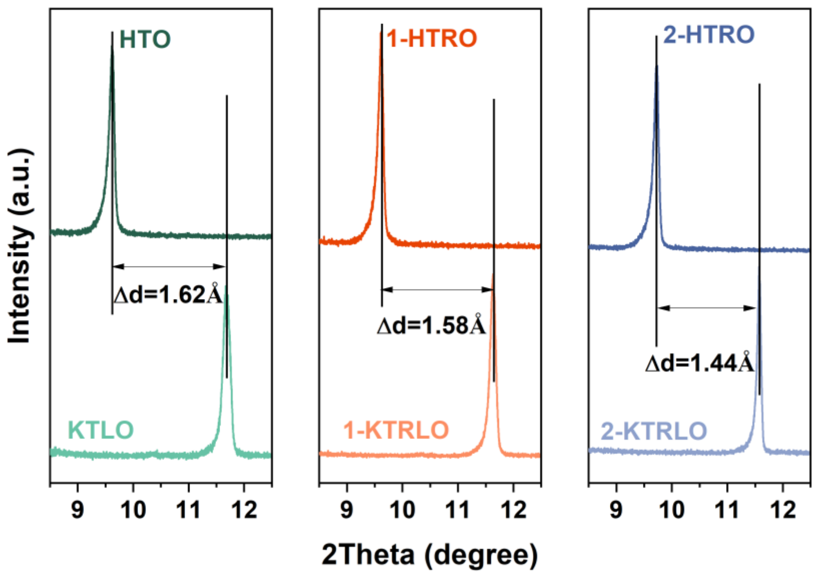

Figure S2. XRD analysis with zoomed in (020) peaks of initial (KTLO,1-KTRLO,2-KTRLO) and protonated (HTO,1-HTRO,2-HTRO) powders.

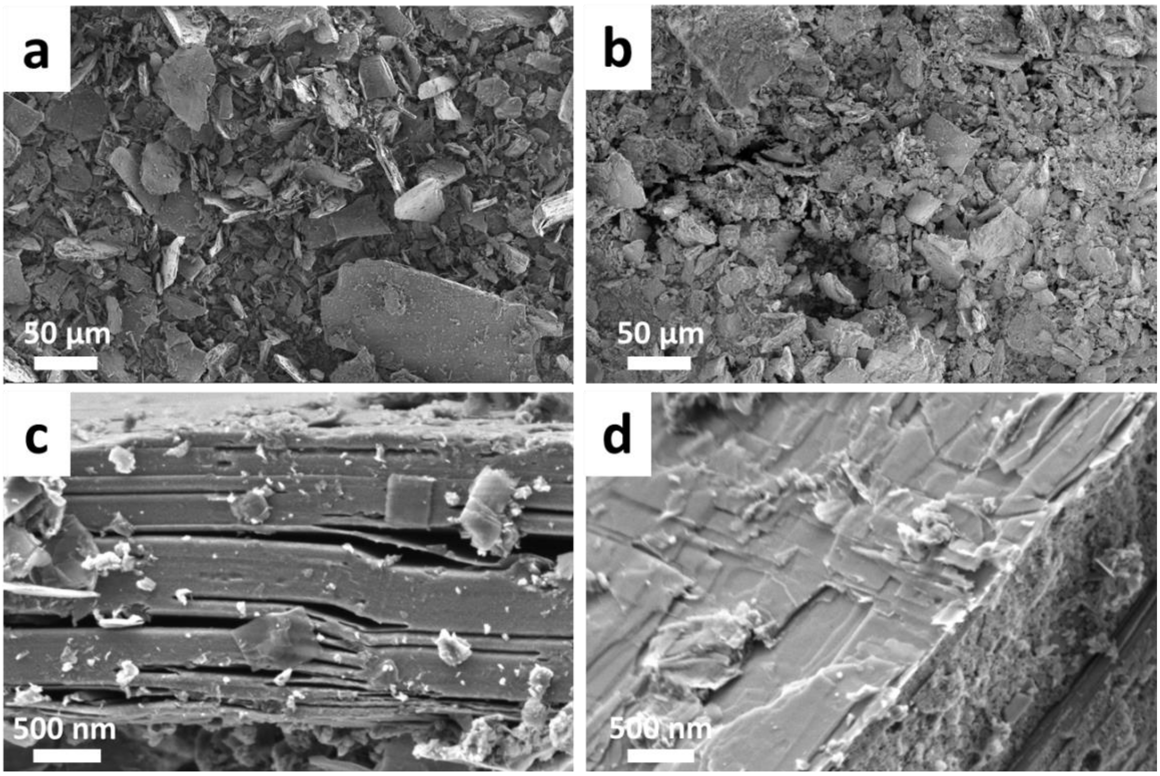

Figure S3. SEM images of (a, c) TO and (b, d) 1-RTO powder particles.

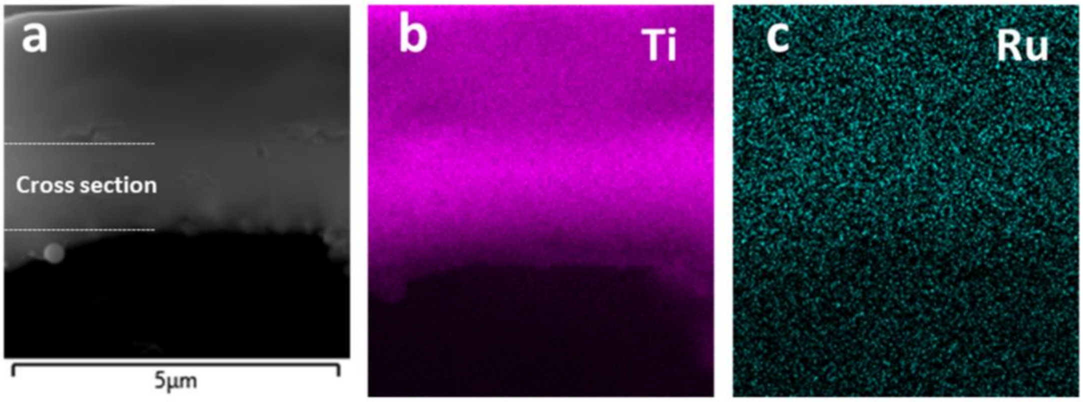

Figure S4. (a) SEM image of 1-RTO particle and the corresponding EDX maps of (b) Ti and (c) Ru.

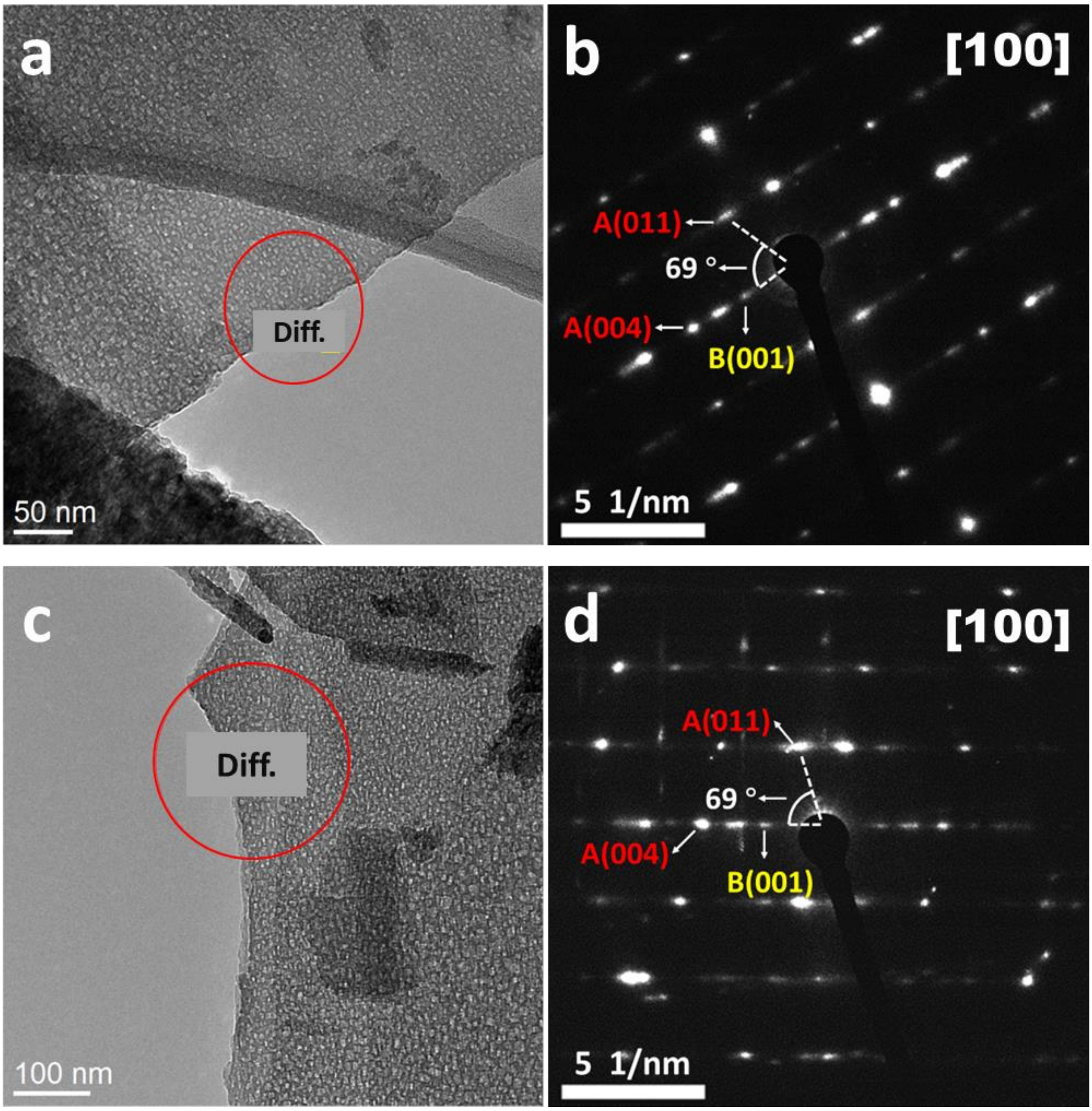

Figure S5. TEM images of (a) TO and (c) 1-RTO particles with selected area for diffraction and corresponding SAED patterns of (b) TO and (d) 1-RTO particles.

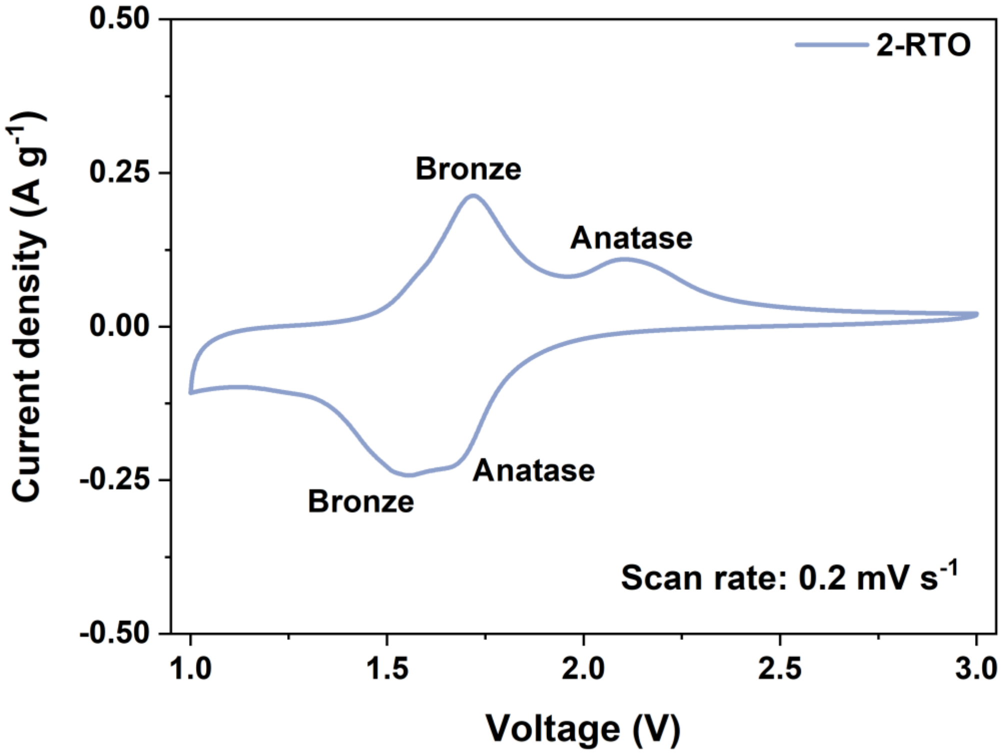

Figure S6. CV curve of 2-RTO based electrode at a sweep rate of 0.2 mV s^{-1}.

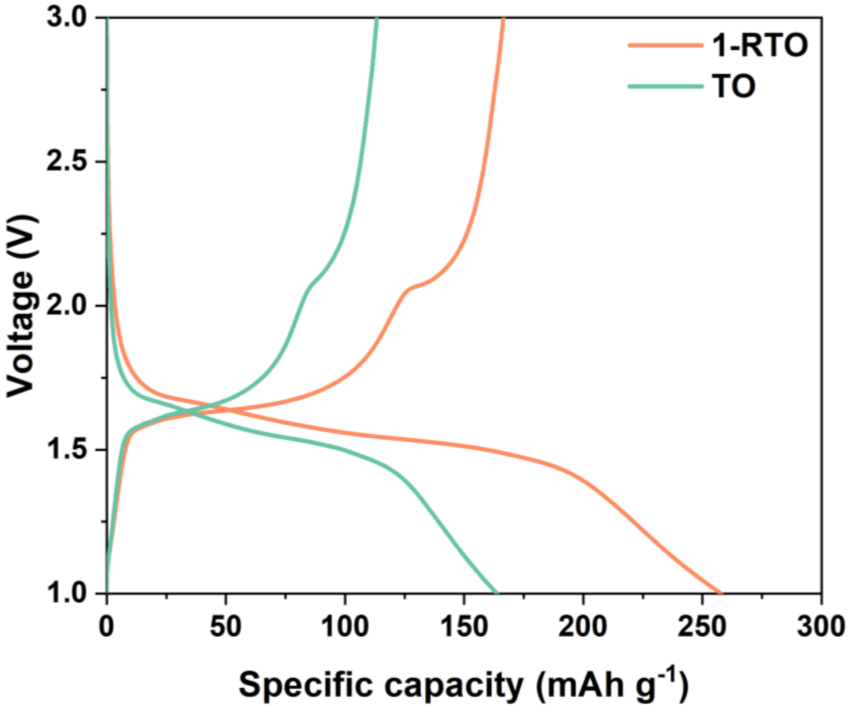

Figure S7. The charge-discharge curves of TO and 1-RTO based electrodes of the first cycle.

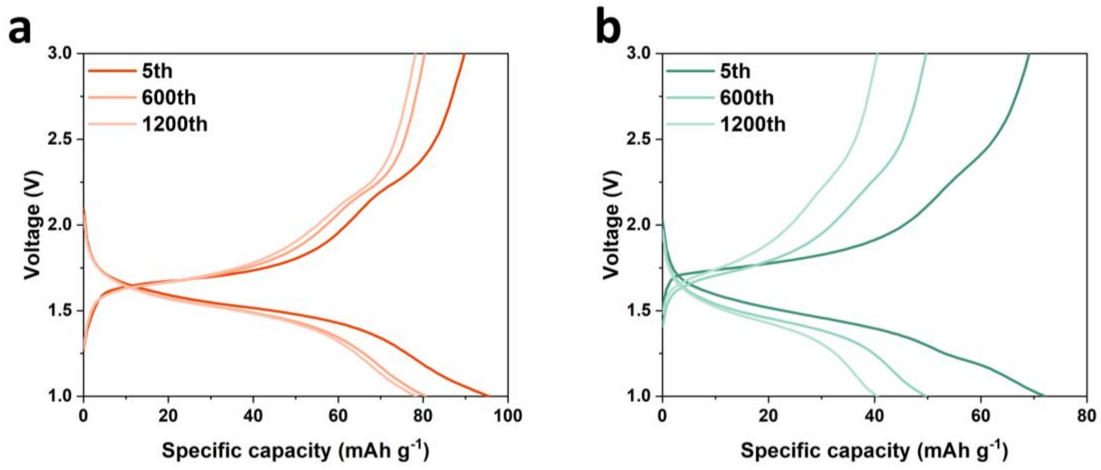

Figure S8. Charge and discharge curves of (a) 1-RTO and (b) TO based electrodes after 5th, 600th and 1200th cycle.
